# Supplementary material for: Performance characteristics and operational feasibility assessment of a CRISPR based tata MD CHECK diagnostic test for SARS-CoV-2 (COVID-19)
Source: PLoS One. 2023 Sep 14;18(9):e0291269. doi: 10.1371/journal.pone.0291269 (PMC10501677; doi:10.1371/journal.pone.0291269)
Supplement: S1 File — (PDF) [file pone.0291269.s001.pdf]

CRISPR Manuscript - Supplementary data

1. Material and Methods

- **TMC-CRISPR results interpretation:**

The interpretation of TMC-CRISPR test results followed the manufacturer's guidelines, as depicted in Figure S1. The control and test bands' intensity were manually read and reported during the manual reading process. However, when using the Tata MD CHECK App, the band intensity formed on the lateral flow strip (LFS) was automatically read by the App, and the results were interpreted as positive or negative using AI logic.

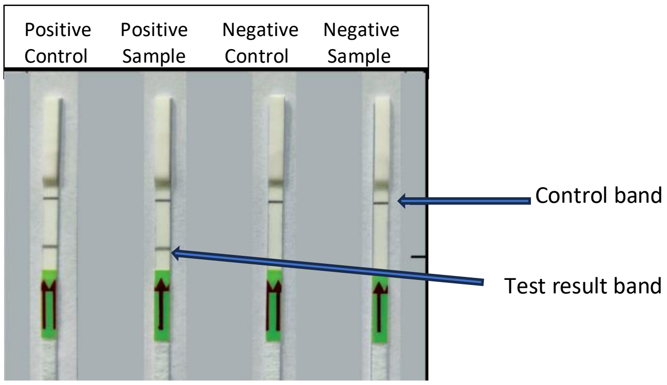

Figure S1 – result strip showing control band, positive and negative results

Examples of results interpreation

**Samples**	**Control Band**	**Test Band**	***Inference***
Positive Control	Present	Present	CRISPR Assay is acceptable
Negative Control	Present	Absent	CRISPR Assay is acceptable
Clinical sample	Present	Present	SARS-CoV-2 positive
Clinical Sample	Present	Absent	SARS-CoV-2 negative
Positive control	Present	Absent	Positive Control degraded
Negative control	Present	Present	Negative control is contaminated
Clinical Sample	Absent	Absent	Storage of strip cards at inappropriate conditions
Clinical Sample	Present	Faint test band seen in all RNA samples	Low level contamination of reagents, cross contamination, repeat the tests

Table S1 – Manual results interpretation based on manufacturer's recommendations. Note: Positive control is used as a indicator or amplification in a batch. There was no internal sample processing control to cross verify amplification of each sample.
