# Supplementary material for: Performance characteristics and operational feasibility assessment of a CRISPR based tata MD CHECK diagnostic test for SARS-CoV-2 (COVID-19)
Source: PLoS One. 2023 Sep 14;18(9):e0291269. doi: 10.1371/journal.pone.0291269 (PMC10501677; doi:10.1371/journal.pone.0291269)
Supplement: S2 File — (PDF) [file pone.0291269.s002.pdf]

CRISPR Manuscript - Supplementary data

1. **Results**

- **Analysis of TMC-CRISPR assay sensitivity and specificity using ICMR guidelines**

Furthermore, we conducted an analysis of the TMC-CRISPR assay's sensitivity and specificity using the Cycle threshold (Ct) cut-off recommended by ICMR, which is set at >35. This assessment was undertaken to validate whether there were any enhancements in the test results under this criterion.

a) Removing E gene ct values > 35   N = 25 removed.

As described in the table S2(a) quick analysis removing 25 samples which had ct values > 35 provided the below results. Sensitivity changes from 44% to 47%

		RT PCR				
		Positive	Negative			
**CRISPR Manual**	Positive	112	25		**Sn**	**47.5%**
	Negative	124	2043		**SP**	**98.8%**
	Total	**236**	**2068**			
**CRISPR app based**	**Row Labels**	Positive	Negative			
	Positive	109	26		**SN**	**47.8%**
	Negative	119	2022		**SP**	**98.7%**
	Total	**228**	**2048**			

Table S2(a): Analysis of TMC-CRISPR results compared to ICMR recommended cut off, Ct >35 for E gene target in the Seegene assay)

b) **Removing N gene ct values > 35   N = 26 removed**

Similar results obtained after removing samples with Ct values of N gene >35

		RT PCR				
		Positive	Negative			
**CRISPR Manual**	Positive	111	25		**Sn**	**47.2%**
	Negative	124	2043		**Sp**	**98.8%**
	Total	**235**	**2068**			
**CRISPR app based**		Positive	Negative			
	Positive	108	26		**Sn**	**47.6%**
	Negative	119	2022		**Sp**	**98.7%**
	Total	**227**	**2048**			

Table S2(b): Analysis of TMC-CRISPR results compared to ICMR recommended cut off, Ct >35 for N gene target in the Seegene assay)

Please note that between the 25 samples in S2(a) and 26 samples in S2(b), only 12 samples are overlapping.
