# Supplementary material for: Performance characteristics and operational feasibility assessment of a CRISPR based tata MD CHECK diagnostic test for SARS-CoV-2 (COVID-19)
Source: PLoS One. 2023 Sep 14;18(9):e0291269. doi: 10.1371/journal.pone.0291269 (PMC10501677; doi:10.1371/journal.pone.0291269)
Supplement: S3 File — (PDF) [file pone.0291269.s003.pdf]

CRISPR Manuscript - Supplementary data

- **Analysis of Ct values for 3 different targets (E, N and RdRp)**

Of the all positive (n=261) samples had a positive Ct value for E (range:14 to 40) and N gene (13 to 40) both. Distributions are given below. However, the RdRp had variations. 206 samples had an Rdrp value reported and the values ranged from 0 to 43. Three samples had 0 reported on ct value for rdrp (Please see the distribution below. The 3 samples that had 0 value reported on rdrp had 33. 35 and 35 reported for E gene and 38,36 and 36 reported on N gene.

In the categorical analysis, for E and G, I have categorised as <=20, 21 to 25, 25 to 30, 31 to 35 and >35. If we go by the same categorization for RdRp, there is marked diff between RdRp and the other 2 targets in the <20, 21-25 and majorly the >35 categories (Please see table below). RdRp was found to be less sensitive than E and N genes in detecting SARS-CoV-2. It could be because of PCR optimised conditions in a multiplex system favours E gene amplification.

	Ct E gene		CT N gene		RdRP gene	
	N	%	N	%	N	%
<20	32	12.26	28	10.73	14	6.8
21-25	50	19.16	49	18.77	18	8.74
26 to 30	77	29.5	73	27.97	56	27.18
31 to 35	77	29.5	85	32.57	56	27.18
>=36	25	9.58	26	9.96	62	30.1
**Total**	**261**	**100**	**261**	**100**	**206**	**100**

Table S3: Analysis of Ct values among different target genes

- **Distribution E gene**

CT-E GENE	Freq.	Percent	Cum.
14	1	0.38	0.38
15	6	2.30	2.68
16	2	0.77	3.45
17	7	2.68	6.13
18	5	1.92	8.05
19	5	1.92	9.96
20	6	2.30	12.26
21	8	3.07	15.33
22	11	4.21	19.54
23	10	3.83	23.37
24	8	3.07	26.44
25	13	4.98	31.42
26	18	6.90	38.31
27	11	4.21	42.53
28	18	6.90	49.43
29	12	4.60	54.02
30	18	6.90	60.92
31	13	4.98	65.90
32	14	5.36	71.26
33	19	7.28	78.54
34	18	6.90	85.44
35	13	4.98	90.42

36	4	1.53	91.95
37	10	3.83	95.79
38	6	2.30	98.08
39	3	1.15	99.23
40	2	0.77	100.00
Total	261	100.00	

- **Distribution N gene**

CT-N GENE	Freq.	Percent	Cum.
13	1	0.38	0.38
14	1	0.38	0.77
15	6	2.30	3.07
16	2	0.77	3.83
17	7	2.68	6.51
18	3	1.15	7.66
19	3	1.15	8.81
20	5	1.92	10.73
21	7	2.68	13.41
22	6	2.30	15.71
23	9	3.45	19.16
24	8	3.07	22.22
25	19	7.28	29.50
26	15	5.75	35.25

27 |	11	4.21	39.46
28 |	24	9.20	48.66
29 |	13	4.98	53.64
30 |	10	3.83	57.47
31 |	20	7.66	65.13
32 |	12	4.60	69.73
33 |	21	8.05	77.78
34 |	20	7.66	85.44
35 |	12	4.60	90.04
36 |	13	4.98	95.02
37 |	5	1.92	96.93
38 |	5	1.92	98.85
39 |	2	0.77	99.62
40 |	1	0.38	100.00
Total |	261	100.00	

- **Distribution RdRp gene**

CT-RDrP |	Freq.	Percent	Cum.
0 |	3	1.46	1.46
18 |	2	0.97	2.43
19 |	1	0.49	2.91
20 |	8	3.88	6.80
21 |	4	1.94	8.74
22 |	3	1.46	10.19
23 |	3	1.46	11.65
24 |	2	0.97	12.62
25 |	6	2.91	15.53
26 |	7	3.40	18.93
27 |	8	3.88	22.82
28 |	11	5.34	28.16

29	8	3.88	32.04
30	22	10.68	42.72
31	13	6.31	49.03
32	11	5.34	54.37
33	11	5.34	59.71
34	9	4.37	64.08
35	12	5.83	69.90
36	15	7.28	77.18
37	11	5.34	82.52
38	13	6.31	88.83
39	9	4.37	93.20
40	12	5.83	99.03
41	1	0.49	99.51
43	1	0.49	100.00
Total	206	100.00	
