# Supplementary material for: Performance characteristics and operational feasibility assessment of a CRISPR based tata MD CHECK diagnostic test for SARS-CoV-2 (COVID-19)
Source: PLoS One. 2023 Sep 14;18(9):e0291269. doi: 10.1371/journal.pone.0291269 (PMC10501677; doi:10.1371/journal.pone.0291269)
Supplement: S4 File — (PDF) [file pone.0291269.s004.pdf]

CRISPR Manuscript - Supporting files

Characteristics	COVIDsure	Seegene-AllplexTM 2019-nCoV Assay	CRISPR (TataMD CHECKTM)
**Purpose**	To generate routine lab reports to patients	Primary comparator	Index test (CRISPER based)
**ICMR approval**	Yes	Yes	Yes
**Target genes**	nCoV ORF1ab (O) and E	E, N and RdRp	S
**Limit of Detection**	~100 copies per reaction	~50-100 copies per reaction	*10-100 copies per reaction (under controlled lab conditions)<br>*~ ≥500 copies per reaction (in real field scenario)

Table S4: Target genes and limit of detection of RT-PCR tests

As per information published in the instruction manual by the manufacturers, the limit of detection of the CRISPR assay is 10-100 copies of the target genetic sequence, when performed under controlled laboratory conditions by well-trained personnel. Under real life diagnostic laboratory conditions, it may be as high as 500-1000 copies of the viral genome. (Ref 6 in the main manuscript reference section).
