# Supplementary material for: Performance characteristics and operational feasibility assessment of a CRISPR based tata MD CHECK diagnostic test for SARS-CoV-2 (COVID-19)
Source: PLoS One. 2023 Sep 14;18(9):e0291269. doi: 10.1371/journal.pone.0291269 (PMC10501677; doi:10.1371/journal.pone.0291269)
Supplement: S5 File — (PDF) [file pone.0291269.s005.pdf]

TataMD CHRCK Supplementary data:

1. Analysis of TMC-CRISPR assay sensitivity and specificity using ICMR suggested cut off of 35 to see if there is any improvement in the results.

A) Removing E gene ct values > 35 N = 25 removed.

A quick analysis removing 25 samples which had ct values > 35 provided the below results. Sn changes from 44% to 47%

		RT PCR				
		Positive	Negative			
**CRISPR Manual**	Positive	112	25		**Sn**	**47.5%**
	Negative	124	2043		**SP**	**98.8%**
	Total	**236**	**2068**			
**CRISPR app based**	**Row Labels**	Positive	Negative			
	Positive	109	26		**SN**	**47.8%**
	Negative	119	2022		**SP**	**98.7%**
	Total	**228**	**2048**			

B) **Removing N gene ct values > 35 N = 26 removed**
Similar results when removing samples with Ct values of N gene >35.

		RT PCR				
		Positive	Negative			
**CRISPR Manual**	Positive	111	25		**Sn**	**47.2%**
	Negative	124	2043		**Sp**	**98.8%**
	Total	**235**	**2068**			
**CRISPR app based**		Positive	Negative			
	Positive	108	26		**Sn**	**47.6%**
	Negative	119	2022		**Sp**	**98.7%**
	Total	**227**	**2048**			

Please note that between the 25 samples in A and 26 samples in B, only 12 samples are overlapping. Rest all samples are different. This could be another reason to go with Kit cut offs. If not we will have to present all tables for N and E gene separately even though Sn and Sps are not different.

2. **Analysis of Ct values for 3 different targets (E, N and RdRp)**

Of the all positive (n=261) samples had a positive Ct value for E (range:14 to 40) and N gene (13 to 40) both. Distributions are given below. However, the RdRp had variations. 206 samples had an Rdrp value reported and the values ranged from 0 to 43. Three samples had 0 reported on ct value for rdrp (Please see the distribution below. The 3 samples that had 0

value reported on rdrp had 33. 35 and 35 reported for E gene and 38,36 and 36 reported on N gene.

In the categorical analysis, for E and G, I have categorised as <=20, 21 to 25, 25 to 30, 31 to 35 and >35. If we go by the same categorization for RdRp, there is marked diff between RdRp and the other 2 targets in the <20, 21-25 and majorly the >35 categories (Please see table below). RdRp was found to be less sensitive than E and N genes in detecting SARS-CoV-2. It could be because of PCR optimised conditions in a multiplex system favours E gene amplification.

	Ct E gene		CT N gene		RdRP gene	
	N	%	N	%	N	%
<20	32	12.26	28	10.73	14	6.8
21-25	50	19.16	49	18.77	18	8.74
26 to 30	77	29.5	73	27.97	56	27.18
31 to 35	77	29.5	85	32.57	56	27.18
>=36	25	9.58	26	9.96	62	30.1
**Total**	**261**	**100**	**261**	**100**	**206**	**100**

Distribution E gene

```
CT-E GENE |      Freq.     Percent        Cum.

------------+-----------------------------------

        14 |          1        0.38        0.38

        15 |          6        2.30        2.68

        16 |          2        0.77        3.45

        17 |          7        2.68        6.13

        18 |          5        1.92        8.05

        19 |          5        1.92        9.96

        20 |          6        2.30       12.26

        21 |          8        3.07       15.33

        22 |         11        4.21       19.54

        23 |         10        3.83       23.37

        24 |          8        3.07       26.44
```

25	13	4.98	31.42
26	18	6.90	38.31
27	11	4.21	42.53
28	18	6.90	49.43
29	12	4.60	54.02
30	18	6.90	60.92
31	13	4.98	65.90
32	14	5.36	71.26
33	19	7.28	78.54
34	18	6.90	85.44
35	13	4.98	90.42
36	4	1.53	91.95
37	10	3.83	95.79
38	6	2.30	98.08
39	3	1.15	99.23
40	2	0.77	100.00
Total	261	100.00	

Distribution N gene

CT-N GENE	Freq.	Percent	Cum.
13	1	0.38	0.38
14	1	0.38	0.77
15	6	2.30	3.07
16	2	0.77	3.83

17	7	2.68	6.51
18	3	1.15	7.66
19	3	1.15	8.81
20	5	1.92	10.73
21	7	2.68	13.41
22	6	2.30	15.71
23	9	3.45	19.16
24	8	3.07	22.22
25	19	7.28	29.50
26	15	5.75	35.25
27	11	4.21	39.46
28	24	9.20	48.66
29	13	4.98	53.64
30	10	3.83	57.47
31	20	7.66	65.13
32	12	4.60	69.73
33	21	8.05	77.78
34	20	7.66	85.44
35	12	4.60	90.04
36	13	4.98	95.02
37	5	1.92	96.93
38	5	1.92	98.85
39	2	0.77	99.62
40	1	0.38	100.00

Total	261	100.00	

Distribution RdRp gene

CT-RDrP	Freq.	Percent	Cum.
0	3	1.46	1.46
18	2	0.97	2.43
19	1	0.49	2.91
20	8	3.88	6.80
21	4	1.94	8.74
22	3	1.46	10.19
23	3	1.46	11.65
24	2	0.97	12.62
25	6	2.91	15.53
26	7	3.40	18.93
27	8	3.88	22.82
28	11	5.34	28.16
29	8	3.88	32.04
30	22	10.68	42.72
31	13	6.31	49.03
32	11	5.34	54.37
33	11	5.34	59.71
34	9	4.37	64.08
35	12	5.83	69.90
36	15	7.28	77.18
37	11	5.34	82.52
38	13	6.31	88.83
39	9	4.37	93.20
40	12	5.83	99.03
41	1	0.49	99.51
43	1	0.49	100.00
Total	206	100.00	
