# Supplementary material for: Performance characteristics and operational feasibility assessment of a CRISPR based tata MD CHECK diagnostic test for SARS-CoV-2 (COVID-19)
Source: PLoS One. 2023 Sep 14;18(9):e0291269. doi: 10.1371/journal.pone.0291269 (PMC10501677; doi:10.1371/journal.pone.0291269)
Supplement: Attachment — Submitted filename: Response to reviewers revised.docx [file pone.0291269.s006.docx]

23^rd^ August 2023

To,

Dr Ruslan Kalendar
Academic Editor
PLOS ONE

We express our gratitude to the academic editor and both the reviewers for their valuable comments and insightful recommendations. We have carefully considered each comment and incorporated necessary revisions into the attached revised version of this manuscript. Below, we provide a detailed response to each comment:

**Response to comments received from Academic Editor: -**

1. Please ensure that your manuscript meets PLOS ONE's style requirements, including those for file naming. The PLOS ONE style templates can be found at

https://journals.plos.org/plosone/s/file?id=wjVg/PLOSOne_formatting_sample_main_body.pdf and

<https://journals.plos.org/plosone/s/file?id=ba62/PLOSOne_formatting_sample_title_authors_affiliations.pdf>

***Response:*** Thank you for reminding us to recheck PLOS ONE’s style requirements including file naming pattern. We tried our level best to make necessary corrections as per PLOS ONE style.

2. We note that the grant information you provided in the ‘Funding Information’ and ‘Financial Disclosure’ sections do not match.

When you resubmit, please ensure that you provide the correct grant numbers for the awards you received for your study in the ‘Funding Information’ section.

***Response:*** Thank you highlighting this important variation. Corrections are made in the “Funding Information” and the correct statement is as mentioned below.

**Funding Information:** This work was supported, in whole or in part, by the Bill & Melinda Gates Foundation #INV-025422. Under the grant conditions of the Foundation, a Creative Commons Attribution 4.0 Generic License has already been assigned to the Author Accepted Manuscript version that might arise from this submission.

3. Thank you for stating the following in the Acknowledgments Section of your manuscript:

"This work was supported, in whole or in part, by the Bill & Melinda Gates Foundation [INV-025422]. Under the grant conditions of the Foundation, a Creative Commons Attribution 4.0 Generic License has already been assigned to the Author Accepted Manuscript version that might arise from this submission. Our sincere thanks to NIRMAN team for all their help and support in conducting feasibility assessment."

We note that you have provided funding information that is not currently declared in your Funding Statement. However, funding information should not appear in the Acknowledgments section or other areas of your manuscript. We will only publish funding information present in the Funding Statement section of the online submission form. Please remove any funding-related text from the manuscript and let us know how you would like to update your Funding Statement. Currently, your Funding Statement reads as follows:

"The authors received no specific funding for this work."

***Response:***

We sincerely appreciate your valuable suggestion. As per your advice, we have taken the necessary steps to remove all funding-related information and text from the acknowledgement section and other parts of the manuscript.

For online publishing purposes, we hereby declare that the project was supported by funding from the Bill & Melinda Gates Foundation [INV-025422] and correct Funding statement is as mentioned below:-

This work was supported, in whole or in part, by the Bill & Melinda Gates Foundation #INV-025422. Under the grant conditions of the Foundation, a Creative Commons Attribution 4.0 Generic License has already been assigned to the Author Accepted Manuscript version that might arise from this submission.

4. In your Data Availability statement, you have not specified where the minimal data set underlying the results described in your manuscript can be found. PLOS defines a study's minimal data set as the underlying data used to reach the conclusions drawn in the manuscript and any additional data required to replicate the reported study findings in their entirety. All PLOS journals require that the minimal data set be made fully available. For more information about our data policy,

***Response:*** We will share a minimum set of data. The PDF version of the data file is attached as a supporting document.

5. PLOS requires an ORCID iD for the corresponding author in Editorial Manager on papers submitted after December 6th, 2016. Please ensure that you have an ORCID iD and that it is validated in Editorial Manager.
6. PLOS requires an ORCID iD for the corresponding author in Editorial Manager on papers submitted after December 6th, 2016. Please ensure that you have an ORCID iD and that it is validated in Editorial Manager. To do this, go to ‘Update my Information’ (in the upper left-hand corner of the main menu), and click on the Fetch/Validate link next to the ORCID field. This will take you to the ORCID site and allow you to create a new iD or authenticate a pre-existing iD in Editorial Manager. Please see the following video for instructions on linking an ORCID iD to your Editorial Manager account: https://www.youtube.com/watch?v=_xcclfuvtxQ

***Response to 6 & 7:*** My ORCID ID details are <http://Orcid.org/0000-0002-7772-8695>

I attempted to follow the instructions to update my information, but I encountered a consistent issue. I am receiving message that “an identical, validated Orchid already exists in the database”. Despite multiple attempts, I continue to receive this same message. I have taken the necessary steps and provided my ORCID ID. Also, cross-verified by logging into my ORCID account and it shows access to PLOSONE. Hence, I kindly request that you commence the review process for the revised documents instead of waiting for the resolution of this ORCID ID issue.

7. Please include a copy of Table 7 which you refer to in your text on page 12.

***Response:*** It is supplementary table S4 not table 7. Corrections are made in the discussion.

8. Please include captions for your Supporting Information files at the end of your manuscript, and update any in-text citations to match accordingly. Please see our Supporting Information guidelines for more information:

***Response:*** We have made all the recommended changes in the revised version of this manuscript. Figure 1 meets PLOS requirements (confirmed using PACE digital tool).

9. We note that several of your files are duplicated on your submission. Please remove any unnecessary or old files from your revision, and make sure that only those relevant to the current version of the manuscript are included.

***Response:*** Old and unnecessary files are removed, and only relevant files are uploaded while submitting revised versions

10. Please amend your current ethics statement in methods section to include the full name of the ethics committee/institutional review board(s) that approved your specific study.

***Response:*** Full name of the ethics committee from KEM hospital is included in the revised version.

11. In the Methods section please include the informed consent statement to reflect whether "written or verbal" informed consent was obtained from all participants for inclusion in the study.

***Response:*** Corrections made as recommended.

**Response to comments received from Reviewers: -**

- **Reviewer # 1**

***# Comment # 1***

a. In general, the table of sensitivity, specificity, positive predictive value and negative predictive value of their test should be in the main file, rather than the Supplemental. It is more informative than % samples and quite important when evaluating the given performance of a test.
b. Can the authors provide the thresholds under which different tests are approved according to their healthcare system? They may change depending on their use (e.g. clinical diagnostics vs population screening; PCR vs lateral flow).

***Response:***

a. Thank you for the feedback. We have revised the table 2 in the main file which presents estimates and 95% CIs for sensitivity, specificity, positive predictive value and negative predictive value for TATA MD (manual) and TATA MD (app based) readings.
b. Validation of SARS-COV2 diagnostic commodities: ICMR acceptance Criteria is described below

| RT-PCR Kit | Sensitivity: 95% and above  Specificity: 99% and above |
| --- | --- |
| RNA Extraction Kit | At least 95% concordance among positive  At least 90% concordance among negative samples  > 95 % samples showing amplification in internal control |
| VTM | 100% concordance among spiked samples  100% samples showing amplification in internal control |
| Antibody Rapid Kit | Sensitivity: 90% and above  Specificity: 99% and above |
| ELISA / CLIA Kit | IgM: Sensitivity- 90% and above  Specificity- 99% and above  IgG: Sensitivity- 90% and above  Specificity- 95% and above |

**Notes:**

1. The cut-offs have been decided after deliberation in ICMR Expert Group with the Drug Controller General of India (DCGI).
2. In case of TMC-CRISPR, manufacturer’s claimed sensitivity of 96.1% and specificity of 98.6% on 149 extracted RNA samples (77 positive, 72 negative) at a reputed 3rd party molecular biology lab and research institute (Reference 6 main manuscript).

***# Comment # 2***

**Line 51:** the description of TMC-CRISPR includes ‘reverse transcription PCR’ – I would highlight that the main difference with standard real time PCR is how the detection of the amplified product is done. At the beginning of the paper, the main premise of using a lateral flow type test is that RT-PCR is complex and expensive to perform.

***Response***

We appreciate the reviewer for bringing up this important point. The TMC-CRISPR assay was initially designed and promoted as a potential replacement for RT-PCR, with the advantage of being less complex and more suitable for field use especially in remote locations in the country. The key features highlighted were the ability to work without the need for an RT-PCR machine and a unidirectional work setup, making it a potentially cost-effective and portable option for COVID-19 testing. However, upon close examination of the assay's principles and work process, it became apparent that further evaluation of its feasibility in a real-world RT-PCR laboratory setup was necessary. The decision to conduct the feasibility evaluation in an RT-PCR laboratory setting was driven by the need to thoroughly assess the assay's performance and reliability, particularly in a controlled and well-established diagnostic environment.

***# Comment # 3***

Ct values are difficult to interpret since thresholds vary from lab to lab – do the authors have an idea of how many copies per mL do Ct = 20 correspond to?

***Response:***

We agree with the reviewer’s comment that there is always slight variation in Ct values as thresholds vary from lab to lab. Further, to determine the number of copies per mL corresponding to a Ct value of 20, we need a standard curve or a known reference sample with a known concentration of the target nucleic acid. We have not done standard curve analysis as that was not the aim for this study. Kit validation was carried out using known positive and negative samples. However, looking at the limit of detection of Seegene RT -PCR, and considering 3-4 Ct difference = 1 log difference, Ct = 20 might be ~ ≥10^6-7^ copies (10,00,000 -1,00,00,000 copies) per reaction.

***# Comment # 4***

Lines 260-261 and 271 appear to contradict each other. Did the participants change or not change their opinion? Does line 271 relate to interpretation of the data? Please clearly state so, there were shifts in opinions as explained by the authors, which are important findings.

***Response***

As outlined in table 6, the interpretation of TMC-CRISPR test results compared to RT-PCR results was discussed in lines 260-261. Notably, all participants maintained their initial opinions throughout the final assessment, indicating that there were no changes in the outcomes between the initial and final assessments.

Additionally, line 271 pertains to the interpretation of data obtained from the app-based system versus the manual method. Subsequent revisions were made to the manuscript based on the findings from this analysis presented in table 6.

***# Comment # 5***

In Discussion (check typo, Discussions), I am missing SHERLOCK as a pioneer system to detect SARS-CoV-2 using CRISPR.

***Response:***

Thank you for pointing out this typo error. We have addressed the suggested corrections and made the necessary changes.

***# Comment # 6***

Please be consistent about the annotation ‘COVID-19’ and not ‘Covid-19’.

***Response:***

Thank you for bringing this error to our attention. We have carefully reviewed the manuscript and made the necessary corrections to ensure consistency throughout.

***# Comment # 7***

**N=9** is not that a small number for a laboratory – for a survey it may be, so I would put that as a limitation although the data is quite clear.

***Response***

Agree. We highlighted this drawback at the beginning of discussion section.

***# Comment # 8***

Does the TMC-CRISPR assay include a control line? Including a picture of the assay would be quite helpful if possible.

***Response***

Yes, the TMC-CRISPR assay incorporates a control line to ensure proper functionality. To provide clarity on the results interpretation of this assay, we have included a standard picture and table, offering a comprehensive understanding of the outcomes in supporting documents.

***# Comment # 9***

The authors mention sensitivity of copies- is that per mL sample? Lateral flow assays have lower thresholds to be considered under UK regulations. Is that the case? In any case, a Ct of 20 should be in the range of millions of copies per mL. Please refer to your figures and tables when you discuss them.

***Response***

Please note that the limit of detection (LOD) of each assay is mentioned as copies per reaction. We agree with reviewer’s comment that lateral flow assays have lower thresholds and that could be one of the reasons of higher limit of detection and lower sensitivity of the TMC CRISPR assay.

Necessary changes were made referring figures and tables in the text as suggested.

***# Comment # 10***

I am also missing some more literature on the general performance of lateral flow assays.

***Response***

The detection of amplified PCR products using the lateral flow paper strip method is generally considered as a rapid, cost effective and convenient approach to visualize the presence of a specific target sequence in the PCR reaction in low-income settings. The sensitivity of such a paper strip-based lateral flow assays for PCR product detection can vary depending on various factors, including the target analyte, the design of the assay, and the detection method employed etc. There are limited reports where lateral flow paper strip method was utilised for detection of SARS-CoV-2 after amplification of cDNA. Agarwal et. al. utilized loop-mediated isothermal amplification (LAMP) followed by lateral flow–based nucleic acid detection of SARS‑CoV‑2 and reported sensitivity of 77% and LOD of 3.9 x 10^3^ that is much lower that RT-PCR sensitivity (50-100 copies/mL). Relevant reference has been added in the main manuscript.

***# Comment # 11***

Line 316: what does ‘gradient’ mean? Do the authors mean that with higher Ct values per RT-qPCR they observed more false negatives in the TMC-CRISPR? If so, please clearly say so.

***Response***

Gradient meaning a slope (incline or decline trend). Yes. We did observe more false negatives (descending trend) in TMC-CRISPR results with increasing RT-PCR Ct values.

***# Comment # 12***

Line 323: I do not understand the argument about a refrigerated centrifuge for RNA extraction, if the gold standard RTqPCR worked with the RNA extracted without such equipment. Do the authors imply that there is RNA degradation in their samples – or that it is so extensive that the test does not work? There is an RT-PCR step also performed, it is the detection of the amplicons that changes.

***Response:***

In the discussion, it is essential to highlight that RNA extraction from clinical specimens typically necessitates the use of a refrigerated centrifuge. The manufacturer claimed that the test could be utilized in remote locations as it does not require RT-PCR machine; however, it is crucial to consider that such areas might lack access to a refrigerated centrifuge for RNA extraction. Subsequently, performing the test in the field and remote locations might not be feasible, posing a significant challenge to its practical implementation in these settings.

**# Comment # 13**

Line 339: what is ‘TAT’

***Response:***

"TAT" is an abbreviation for "turnaround time." Please refer to line 215 for a detailed explanation where the full form and its corresponding abbreviation are provided.

***# Comment # 14***

I am not sure how ‘extensive validation’ will help in this case. If the test is not sensitive enough, it requires re-designing. As it stands, the authors have shown quite convincingly that it does not work. Is there a possibility that the test was designed and approved for the original variant, and it does not work with the tested variant?

***Response:***

We are sincerely grateful to the reviewer for bringing up this significant aspect. In the case of TMC-CRISPR assay, the manufacturers must undertake a diligent process of assay redesign and development, incorporating improved targets and a more sensitive detection method than LAS. However, the pivotal step following the development phase is to conduct an extensive validation of the assay. This involves testing a substantial number of samples with lower viral loads, approaching the limit of detection (LOD) of the assay. Such thorough validation is indispensable to ensure the assay's effectiveness and suitability for use in real-world field conditions, which can vary considerably.

**Reviewer # 2**

***# Comment # 1***

Table 2. Although the negative results and inconclusive are mentioned in the legends. I propose making a simpler table of TMC-CRISPR vs Seegene RT-PCR showing the positives and negatives in the table.

***Response:***

Thank you for the comment. Details of TMC-CRISPR vs Seegene RT-PCR showing positives, negatives, inconclusive and invalid have already been provided in table 1. We hope this table meets the suggested distribution.

***# Comment # 2***

Study limitation. Consider including the use of different sample types ie nasopharyngeal, throat swab and nasal swab and endotracheal aspirates in the analysis as a potential source of bias since the different sample types may have different performances.

***Response:***

This study encompasses a comprehensive range of sample types, including throat swabs, nasal swabs, nasopharyngeal swabs, and a combination of throat + Nasal swabs and throat + nasopharyngeal swabs, etc. as depicted in figure 1. Our analysis delved deeply into the outcomes based on these distinct sample types and we observed consistent specificity across all types. However, it is noteworthy that sensitivity exhibited some variability. Further, it’s important to mention that the sample size for the combined throat + nasal swabs subset was relatively smaller, rendering the interpretation of a sensitivity score of 100 impractical (not interpretable). As a result, we have omitted this particular aspect from our discussion for the sake of clarity and accuracy.

***# Comment # 3***

Line 322. The authors mention OFA highlights need for unidirectional laboratory setup to perform TMC-CRISPR test. Since this is also required in the RT-PCR I think this point should be excluded from the manuscript.

***Response:***

The response is similar to the one highlighted in response to comment 2 from reviewer 1. We appreciate the reviewer for bringing up this important point. The TMC-CRISPR assay was initially designed and promoted as a potential replacement for RT-PCR, with the advantage of being less complex and more suitable for field use especially in the remote locations in the country. The key features highlighted by the manufacturer were the ability to work without the need for an RT-PCR machine and a unidirectional RT-PCR work setup, making it a potentially cost-effective and portable option for COVID-19 testing. However, upon close examination of the assay's principles and work process, it became apparent that further evaluation of its feasibility in a real-world RT-PCR laboratory setup was necessary. Hence, we think this point is important to discuss in the discussion section.

Please note that there is a correction in Authors name A.S. Thekkepurallar should be written as Akhil S. ThekkePurakkal. Furthermore, it should be noted that authors M. Jinwal and Akhil S. ThekkePurakkal have made an equivalent contribution to this manuscript, thereby sharing equal third authorship. It is important to highlight that this acknowledgment does not alter the established sequence of author listing.

Once again, thank you for your efforts in helping us to improve this manuscript, and we are grateful for your continued support.

Thanks, and Regards

Shubhada

**Dr Shubhada Shenai** I Medical Officer
FIND | Flat No. 8, 9th Floor, Vijaya Building, 17 Barakhamba Road, New Delhi-110001, India

M +91 98 67 96 37 47
